# Effect of Interleukin-29 on Interferon-α Secretion by Peripheral Blood Mononuclear Cells

**DOI:** 10.22074/cellj.2015.497

**Published:** 2015-01-13

**Authors:** Chi Hyun Cho, Soo Young Yoon, Chang Kyu Lee, Chae Seung Lim, Yunjung Cho

**Affiliations:** Department of Laboratory Medicine, College of Medicine, Korea University, Seoul, Korea

**Keywords:** IL-29, IFN-α, CpG, Plasmacytoid Dendritic Cells, Peripheral Blood

## Abstract

**Objective:**

The effect of interleukin (IL)-29, a new therapeutic agent similar to type I interferons (IFNs), on IFN-α secretion of human plasmacytoid dendritic cells (pDCs) has not been studied. Therefore, in this study, we aimed to clarify the effect of IL-29 on IFN-α secretion of pDCs using human peripheral blood mononuclear cells (PBMCs) in the presence of cytosine-phosphate-guanosinemotif-containing oligodeoxy nucleotides (CpG).

**Materials and Methods:**

In this experimental and prospective study, PBMCs were ob- tained from 11 healthy volunteers and divided into four culture conditions: I. control, II. CpG treatment, III. IL-29 treatment and IV. CpG plus IL-29 treatment. The amount of IFN-α secretion was measured from each culture supernatant by flow cytometry using the flowcytomix apparatus (eBioscience, Vienna, Austria). Fractional IFN-α production of the cultured PBMCs was measured by intracellular staining using the cytomics FC 500 system (Beckman Coulter, Brea, CA, USA) with CXP Software.

**Results:**

The mean ± standard deviation (SD) of supernatant IFN-α secretion per pDC/μL was 5.7 ± 9.3 pg/mL/count/µL for condition I, 1071.5 ± 1026.6 pg/mL/count/µL for condition II, 14.1 ± 21.1 pg/mL/count/µL for condition III, and 1913.9 ± 1525.9 pg/mL/count/µL for condition IV. There were statistically significant differences between conditions I and II as well as betweenconditions II and IV. Intracellular IFN-α production was only detectable in the pDC fraction from one culture; the production amount was similar between the cells treated with CpG and those treated with CpG plus IL-29. Natural killer (NK) cell production of IFN-α was observed in two out of three cultures and one culture showed IFN- α production in the monocyte fraction.

**Conclusion:**

IL-29 alone did not show any effect on IFN-α secretion of PBMCs. However, the addition of CpG along with IL-29 enhanced IFN-α secretion of PBMCs. Given that pDCs are the major secretors of IFN-α in peripheral blood, this result has suggested the possibility that IL-29 has an enhancing effect in human pDC IFN-α secretion. Although the supernatant IFN-α secretion was not directly correlated with pDCs’s intracellular IFN-α production in this study, prolonged incubation of pDC and other PB subsets with CpG or IL-29 for over 4 hours could be applied in future studies. These studies would help to elucidate the mechanism of action of IL-29 in human pDCs associated with viral infections.

## Introduction

Plasmacytoid dendritic cells (pDCs) have a central role in linking innate and adaptive immunity (1). PDCs have similar morphology to plasma cells and more abundantly express the endosomal Tolllike receptors (TLRs) 3, 7, 8, 9 that recognize nucleic acids of internalized viruses such as singlestranded RNA (ssRNA), double-stranded RNA (dsRNA), or cytosine-phosphate-guanosinemotifcontaining oligodeoxynucleotides (CpG) (1-6). Recognition of ssRNA via TLR7 and unmethylated CpG via TLR9 results in the secretion of type I interferon (IFN) in pDCs (7). Type I IFN mRNA can be produced as early as 4 hours after viral stimulation, and a large amount of type I IFN (50000-100,000 pg/mL) can be secreted within 24 hours (8).Therefore pDCs are the major source of antiviral cytokine type I IFN that expresses type I interferons in response to viral infection (9).

Human pDCs do not express lineage-specific markers for all known cell types within the immune system (1, 8, 10). They do not express surface and cytoplasmic immunoglobulin, cluster of differentiation (CD)19 (B cells), TCR and CD3 (T cells), CD14 (monocytes), CD16 and CD56 natural killer (NK) cells, or CD11c (myeloid DC) (8). In addition, pDCs do not express myeloid cell markers, such as CD 11b, CD13, CD14, or CD33, nor do they exhibit nonspecific esterase and phagocytic activity (8).

PDCs are identified in primary lymphoid tissues such as the fetal liver, thymus, and bone marrow (8). During adult life, pDCs appear to be continually produced from bone marrow (8). Starting from bone marrow, pDCs migrate into the T cell-rich areas of the secondary lymphoid tissues through high endothelial venules in lymph nodes (1, 5). pDCs represent 0.2-0.8% of peripheral blood mononuclear cells (PBMCs) in humans (10).

Interleukin (IL)-29 is a type III IFN (IFN-λ), which is called IFN-λ1. IL-29 binds to IFN-λR1 and IL-10R2 heterodimers (11). The expression of IFN-λR1 in PBMCs is known to be limited to the pDC fraction. It has been reported that monocytes, macrophages, myeloid DCs, T cells, and NK cells do not respond to IL-29 stimulation (11-13).

Unmethylated CpG are present at high frequencies in viruses and bacteria; unmethylated synthetic CpG stimulate pDCs via TLR9 (4, 7). The distribution of TLR9 in humans is restricted to pDCs and B cells, so that the antiviral activity of CpG is mainly accomplished by the production of high amounts of IFN-α by pDCs, which in turn affects other immune cells, such as cytotoxic T lymphocytes and NK cells. Among the three classes of CpGs (A, B, and C), CpG A is known to stimulate high amounts of IFN-α production in pDCs and only weakly stimulates DC maturation (4).

Although the modulation of human pDCs by IL- 29 or CpG has been reported previously (13-15) the effect of IL-29 on IFN-α secretion of human pDCs has not been studied thus far. Therefore we aimed to clarify the effect of IL-29 with or without CpG on IFN-α secretion in human pDCs using PBMCs.

## Materials and Methods

### Subjects

In this experimental and prospective study, we recruited 11 healthy volunteers: 2 males and 9 females with an age range from 23 to 45 years. Subjects did not suffer from any systemic immune diseases, congenital immune deficiencies, cancers, viral and/or bacterial infections, hematological diseases, and/or diabetes, and had no history of immunosuppressive drug treatment, thus fulfilling the criteria described in an earlier study (10). Informed consent was obtained from all volunteers and the study was approved by the Korea University Guro Hospital Internal Review Board (IRB number KUGH12093).

### Isolation and culture of PBMCs

Fresh peripheral blood (PB, 10 mL) from each donor was treated with acid citrate dextrose (ACD), and PBMCs were isolated using the Ficoll- Hypaque density gradient (density 1.077; Biochrom, Berlin, Germany) centrifugation method. Isolated PBMCs were resuspended in RPMI 1640 medium (Gibco, Grand Island, NY) that contained 2 mmol/L L-glutamine, 1 mmol/L sodium pyruvate, 100 units/mL penicillin, 100 μg/mL streptomycin, and 5% pooled human AB serum. Pooled human AB sera was obtained from the Korea University Guro Hospital Blood Bank and confirmed to be negative for HBC, HCV, HIV, and syphilis. This sera was heat-inactivated at 56˚C for 30 minutes and filtered with a 2 μm filter (16).

PBMCs from each donor were grown in four different culture conditions: I. control, II. incubated with CpG A (Invitrogen, San Diego, CA, USA) at 2 μM, III. incubated with IL-29 at 500 ng/mL (R&D Systems, Minneapolis, MN, USA) and IV. incubated with CpG A (2 μM) and IL-29 (500 ng/mL). Each culture was maintained in 96-well round bottom plates that contained 0.2 mL of culture media per well at a concentration of 5×10^5^ cells/mL. These plates were incubated for 24 hours in an incubator set at 37˚C and 5% CO_2_. At the end of the incubation period, 150 μL of supernatant was harvested and kept frozen until IFN-α analysis.

### Measurement of IFN-α secretion in culture supernatants (FlowCytomix assay)

Human INF-α FlowCytomix and Human Basic FlowCytomix kits (eBioscience, Vienna, Austria) were used for measuring IFN-α secretion in culture supernatants according to the manufacturer’s instructions. Measured data were analyzed with FlowCytomix Pro 2.1 Software (Bender MedSystems, Vienna, Austria). The analytical measurement range of the Human IFN-α FlowCytomix kit with FlowCytomix Pro 2.1 Software was 0.0– 20000.0 pg/mL. The measured value of IFN-α divided by the pDC count/μL resulted in supernatant IFN-α /pDC number (16).

### Enumeration of pDCs in PBMCs

**Fig 1:**
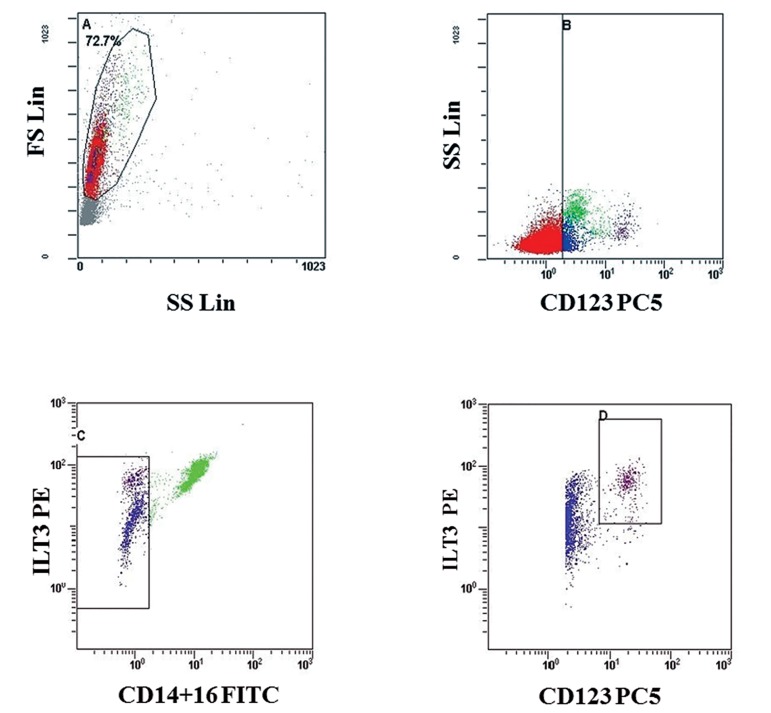
Percentages of plasmacytoid dendritic cells (pDCs) in peripheral blood mononuclear cells (PBMCs) measured by flow cytometry. The A region points to lymphocyte and monocyte regions. In B region, all CD123pos cells were selected. In the C region, ILT3pos cells and CD14+CD16neg cells were gated. As ILT3pos cells (or CD85kpos cells) were gated, all basophils (CD85kneg) were excluded; monocytes and DCs (CD85kpos) were restricted. CD14+CD16neg cells were selected to exclude all monocytes. Then, ILT3pos and CD123pos cells were selected, pointing to the D region.

The percentage of pDCs in PBMCs was obtained by a three-color flow cytometric method within 4 hours of PBMC preparation. A mixture of monoclonal antibodies specifically established to identify pDCs was used. Cells were stained with CD14-FITC, CD16-FITC, CD85k (ILT3)-PE, and CD123-PC5 according to the manufacturer’s instructions (Beckman Coulter, Brea, CA, USA). pDCs were identified as being CD14lo/-CD16lo/- CD85k (ILT3)± and CD123± (10). Briefly, 100 μL of PBMCs were incubated with a mixture of the monoclonal antibodies (20 μL) for 20 minutes at room temperature (RT) in the dark. After two washes with PBS, stained aliquots of PBMCs were reconstituted in 0.5 mL of PBS. Flow cytometry readings of the stained cells were acquired using Cytomics FC 500 (Beckman Coulter, Brea, CA, USA) with CXP Software (Fig 1).

Absolute pDC numbers were estimated by multiplying the percentage of pDCs by the number of PBMCs, which was calculated by multiplying the number of white blood cells (WBCs) by the percentage of lymphocytes plus monocytes in WBCs as measured in the Sysmex XE-2100 apparatus (Sysmex Corporation; Kobe, Japan).

### Intracellular IFN-α assay (ICS assay)

Donors that had evidence of high IFN-α secretion from the PBMC culture supernatant or a high proportion of pDCs in PBMCs were selected. Another set of PBMC cultures were divided into the same conditions as the above in order to perform the ICS assay that included the addition of brefeldin A (Sigma Aldrich, St. Louis, MO, USA) at a final concentration of 10 μg/mL, 4 hours after starting the incubation. The cells were harvested after a 4-hour incubation with brefeldin A (17). Three-color or four-color flow cytometric ICS assays were performed for T-cells, B-cells, NKcells, monocytes and pDCs.

The antibodies used were CD3-PE for T cells, CD19-FITC for B cells, CD14-PE for monocytes and CD56-FITC for NK cells (Beckman Coulter, Brea, CA, USA). CD14-FITC, CD16-FITC, CD85k (ILT3)-PE, and CD123-PC5 antibodies were used for pDCs. Briefly, cells were fixed with IntraPrep fixation agent (Beckman Coulter, Marseille, France) for 15 minutes at RT, and then washed and permeabilized in an IntraPrep permeability agent (Beckman Coulter, Marseille, France) for 5 minutes at RT. After washing, the pellets were stained with PE-Cy7-anti-IFN-α antibody (Bioss, Woburn, MA, USA). Using a Cytomics FC 500 flow cytometer, 200,000-500,000 events were collected and analyzed with CXP software. Gated regions that showed less than 1% of IFN-α-producing cells meant that those cells did not produce IFN-α.

### Statistical analysis

Analyses were performed using SPSS software (version 20.0, SPSS, Chicago, IL, USA). The Friedman test was used to compare the secretion of IFN-α among four culture conditions and p values <0.05 were considered statistically significant. When statistically significant values were observed, Wilcoxon signed rank test was performed between the two groups and p values <0.0083 (=0.05/6) were considered statistically significant.

## Results

### Enumeration of PBMCs pDCs

Table 1 shows the age, sex and frequencies of WBC, lymphocytes, monocytes and pDCs of the 11 study subjects.

**Table 1:** The frequencies of WBCs, lymphocytes, monocytes and pDCs in the peripheral blood from 11 volunteers


Volunteernumbersex/age (Y)	WBC/μL	Lympho (%)	Mono (%)	pDC (%)	pDCs/μL*

**1 F/37**	4190	45.80	5.00	0.23	4.90
**2 F/39**	5570	36.80	9.00	0.49	12.50
**3 F/43**	6000	29.50	6.20	0.33	7.06
**4 M/35**	4360	33.90	6.70	0.21	3.72
**5 F/48**	4370	43.70	7.60	0.13	2.91
**6 F/22**	5910	25.70	3.90	0.23	4.03
**7 F/27**	6130	27.10	5.20	0.41	8.12
**8 F/29**	5950	36.30	7.40	0.09	2.34
**9 F/35**	5880	27.90	5.80	0.30	5.94
**10 F/31**	5070	43.80	5.70	0.10	2.51
**11 M/23**	5250	27.80	7.20	0.19	3.50
**Mean ± SD**	5335 ± 735	34.39 ± 7.44	6.34 ± 1.43	0.25 ± 0.13	5.23 ± 3.05


*; The estimated pDC/uL was calculated by multiplying pDC% with PBMCs [WBC × (lymphocytes% + monocytes%)]. WBCs; White blood cells, Lympho; Lymphocytes, Mono; Monocytes, pDCs; Plasmacytoid dendritic cells, PBMCs; Peripheral blood mononuclear cells and SD; Standard deviation. The data are displayed in the same order as table 2.

### IFN-α secretion in the culture supernatants of PBMCs

The mean ± SD (n=11) of IFN-α concentration per pDC/μL for each culture condition was as follows: condition I. 5.7 ± 9.3 pg/mL/count/μL, condition II. 1071.5 ± 1026.6 pg/mL/count/μL, condition III. 14.1 ± 21.1 pg/mL/count/μL and condition IV. 1913.9 ± 1525.9 pg/mL/count/μL. PBMC cultures that added CpG alone or combined CpG and IL-29 showed significantly increased secretion of IFN-α, whereas the condition with IL-29 alone did not show any significant changes in IFN-α secretion. Co-culture with CpG plus IL-29 stimulated human PBMCs to nearly double the secretion of IFN-α compared to those stimulated by CpG alone (p=0.003, Table 2).

**Fig 2:**
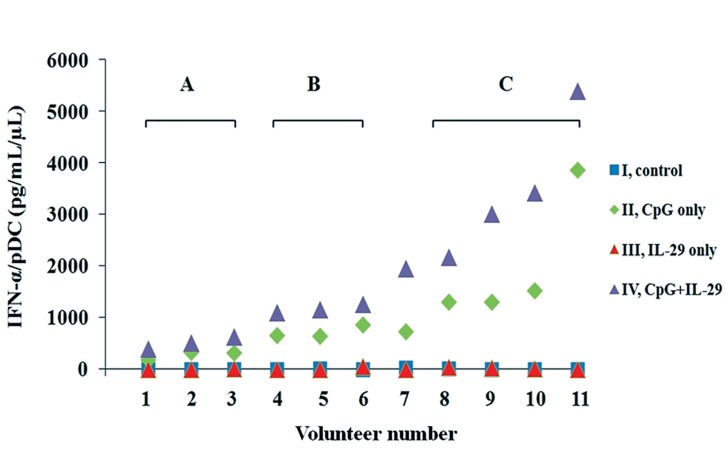
Supernatant IFN-α concentration/plasmacytoid dendritic cell (pDC) in different culture conditions: I. control, II. CpG alone, III. IL-29 alone and IV. CpG and IL-29. Based on increasing IFN-α secretion, three groups were identifiable. A pronounced tendency of increased secretion of IFN-α in response to CpG or CpG and IL-29 (violet triangles) was observed in group C (volunteers 8, 9, 10, 11). Only a slight increase in IFN-α secretion was observed in group B (volunteers 4, 5, 6). A negligible change in IFN-α secretion was noted in group A (volunteers 1, 2, 3). However, regardless of the difference in the amount of IFN-α secretion all volunteers showed approximately double the amount of IFN-α secretion in condition IV compared with condition II.

When we compared the results based on increasing IFN-α secretion, three groups were identifiable: group A that had a minimum to negligible increase (volunteer numbers 1, 2, and 3), group B had a slight increase (volunteer numbers 4, 5, and 6), and group C showed marked increase (volunteer numbers 8, 9, 10 and 11). The response of volunteer number 7 with CpG plus IL-29 was similar to those of group C, in which the CpG plus IL-29 stimulus more than doubled the response caused by the CpG-only stimulus, even though the effect of CpG alone was similar to those of group B (Fig 2).

**Table 2:** Supernatant interferon α (IFN-α) concentration per plasmacytoid dendritic cells (pDCs)/μL under different culture conditions


Volunteernumbersex/age (Y)	I*	II	III	IV	IV/II ratio

**1 F/37**	0.00	195.12	0.00	393.47	2.02
**2 F/39**	0.00	340.90	0.00	513.09	1.51
**3 F/43**	2.68	327.62	8.96	631.82	1.93
**4 M/35**	4.72	656.32	0.00	1108.74	1.69
**5 F/48**	9.42	643.83	0.00	1166.94	1.81
**6 F/22**	0.00	859.50	61.34	1266.66	1.47
**7 F/27**	30.81	738.69	0.00	1952.90	2.64
**8 F/29**	11.75	1310.00	42.72	2178.76	1.66
**9 F/35**	0.00	1310.14	29.69	3010.13	2.30
**10 F/31**	0.00	1532.17	9.79	3426.37	2.24
**11 M/23**	3.05	3872.72	3.05	5403.99	1.40
**Mean ± SD**	5.68 ± 9.28	1071.55 ± 1026.60	14.14 ± 21.11	1913.90 ± 1525.93	1.88 ± 0.39


The data are shown in order of increasing IFN-α concentration. I*; Control, II; CpG alone, III; IL-29 alone and IV; Both CpG and IL-29. There was a statistically significant increase in IFN-α secretion in culture condition IV compared with culture condition II (p=0.003, Wilcoxon signed rank test).

### Intracellular IFN-α production of the each cell fractions of PBMCs

The four volunteers (numbers 2, 3, 7, and 9) who showed a high proportion of pDCs in PBMCs were selected for an ICS assay of T, B, NK cells, and monocyte fractions, although ICS was only successful for three of the four samples (numbers 2, 3, and 7) (Table 1). We did not detect intracellular IFN-α production of B or T cell fractions in response to CpG or IL-29 in any of the three samples. Intracellular IFN-α production in the monocyte fraction in response to IL-29 or CpG plus IL-29 was detected in volunteer number 7, but there was no response to CpG stimulation alone. Intracellular IFN-α production of the NK cell fraction was detected in all three samples; there was a slight tendency of stronger responses to IL-29 compared to CpG, although a statistical significance could not be given due to the small number of samples (Table 3).

**Fig 3:**
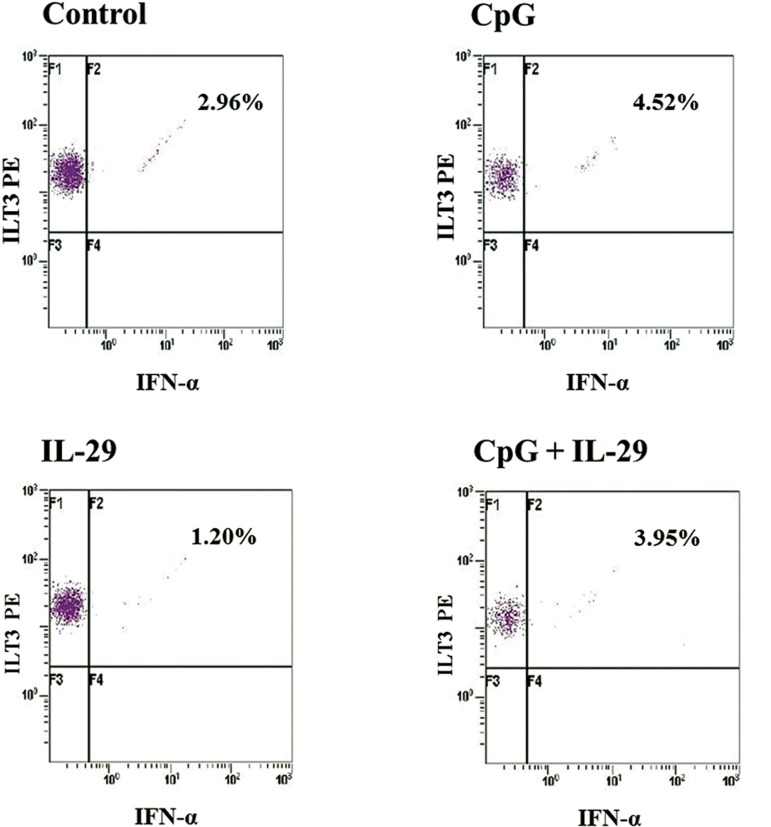
Production of IFN-α in plasmacytoid dendritic cells (pDC) in response to CpG or IL-29 as measured by intracellular flow cytometry. A. I (control). Among cells gated as pDCs, 2.96% of cells produced IFN-α. B. II (CpG alone). The cells that produced IFN-α increased to 4.52% compared to the control. C. III (IL-29 alone). Among cells gated as pDC, 1.20% produced IFN-α. D. IV (CpG and IL- 29). A total of 3.95% of cells produced IFN-α which was similar to peripheral blood mononuclear cells (PBMCs) treated with CpG alone.

Three volunteers (numbers 5, 9, and 10) with high IFN-α secretion in response to CpG or IL-29 in the supernatant assay were selected for an ICS assay of pDCs (Table 2). Only one of the three cultures was successful (volunteer number 10). Intracellular IFN-α productions of the pDC fraction in response to CpG or CpG plus IL-29 stimuli were similar, whereas a slight response to IL-29 was observed (Table 3, Fig 3).

**Table 3:** Percentage of IFN-α ICS-positive cells in each peripheral blood mononuclear cell (PBMC) fraction


Cellular fractions	I*	II	III	IV

**2†. T cells**	0.00	0.76	0.78	0.78
**B cells**	0.00	0.25	0.00	0.00
**NK cells**	0.81	2.61	6.08	4.11
**Mono**	0.18	0.48	0.00	0.59
**3. T cells**	0.00	0.10	0.03	0.02
**B cells**	0.04	0.08	0.10	0.17
**NK cells**	0.00	1.63	1.59	1.66
**Mono**	0.00	0.00	0.14	0.19
**7. T cells**	0.69	0.84	0.46	0.74
**B cells**	0.00	0.00	0.00	0.00
**NK cells**	2.23	6.09	8.53	9.58
**Mono**	2.23	0.90	3.93	4.19
**10. pDCs**	2.96	4.52	1.20	3.95


I*; Control, II; CpG alone, III; IL-29 alone, IV; CpG plus IL-29 and †; 2, 3, 7 and 10 are the numbers that represent specific volunteers.

## Discussion

This study has intended to indirectly determine the effect of IL-29 on the production and secretion of IFN-α by human pDCs using PBMCs. pDCs are known to respond strongly to IL-29 and to produce IFN-α rapidly and vigorously upon stimulation of viruses or synthetic TLR agonists (4, 11).Therefore we have postulated that IL-29 could affect pDC IFN-α secretion with CpG (8). Unmethylated CpG induced a strong immune response in B cells and pDCs through TLR9 during viral infections (14).

In this study, human PBMCs have been chosen as the experimental specimen for several reasons. First, the frequency of pDCs is very low, comprising only approximately 0.2-0.8% of human PB (10). Second, isolating pDCs from human PB is not efficient, and the procedure itself can modify the cellular functions and bias the results of the experiment. Third, there have been reports showing that a crosstalk between the cell components occurs during immune reactions *in vivo* (18, 19).

Therefore, an optimal experimental design would be to achieve conditions as similar to the *in vivo* condition as possible.

There was no statistically significant difference in IFN-α secretion between control and IL-29 (IFN- λ1) alone treatment groups (culture conditions I vs. III). However, groups treated with CpG alone (culture condition II) showed higher IFN-α secretion than the other groups, the control and IL-29 alone treatment group. Groups treated with CpG plus IL-29 (culture condition IV) demonstrated the highest IFN-α secretion. Stimulation with CpG plus IL-29 (culture condition IV) (Table 2) in the supernatant assay showed a consistent result regardless of the specific magnitude of the response: an approximate two-fold increase of IFN-α secretion was observed compared to the response to CpG stimulus alone. Based on these results, we have concluded that IL-29 itself did not induce direct IFN-α secretion of PBMCs, whereas it exerted an indirect influence on IFN-α secretion of PBMCs. Given that pDCs are the major secretors of IFN-α in peripheral blood, this result has suggested the possibility that IL-29 has an enhancing effect in human pDC IFN-α secretion. Since CpGs are pathogen-associated molecular patterns, one can consider IL-29 administration to viral infected subjects to enhance IFN-α secretion of pDCs, ultimately strengthening anti-viral effects of pDCs. A mouse study has demonstrated a strong dependency of anti-viral host defense on IFN-αβ but not on IFN-λ. Ank et al. (20) have suggested the possibility that IFN-λwas normally not essential for mounting sufficient antiviral activity. Our results showed that IL- 29 alone did not induce IFN-α secretion which supported their suggestion.

Similar to IFN-α, IL-29 induces the JAK-STAT antiviral pathway (21). Also, genetic association and *in vitro* studies have proposed an interactive and complementary relationship between IFN-λ and IFN-α (21, 22). Interestingly, the combined effects of IL-29 and IFN-α were primarily additive (23). Our data indicated that IL-29 indirectly enhanced pDC IFN-α secretion by CpG. Possibly IL-29 has regulated the induction of IFN-α through interferon regulatory factor-7 (IRF-7), while IRF- 7 is involved in IFNA gene activation during the late phase of type I IFN induction (11). Given the report that IL-29 induced IRF-7 in pDCs (11, 20), IL-29 could have increased the induction of INF-α at the level of gene transcription.

With CpG as the only stimulus (culture condition II), we were able to identify three groups of responses in IFN-α secretion: group A which had a minimum response (numbers 1, 2, and 3), the slight response group or group B (numbers 4, 5, and 6), and the largest response group which was group C (numbers 8, 9, 10, and 11). The only discernable difference between these three groups were the mean ages: group A, 39.7 years; group B, 33 years and group C, 29.5 years, although this difference did not reach statistical significance - likely due to the small number of specimens. Orsini et al. (10) reported that the number of pDCs significantly reduced with increasing age; however, our donors’ ages were within a relatively stable plateau range, which indicated that the difference observed could not be explained by age variations alone (10). Combining these results with those of the ICS assay of two samples from group A (volunteer numbers 2 and 3) and one sample from volunteer number 7, possibly NK cells might play a role in the response to the CpG stimulus because the amount of IFN-α secretion by PBMCs appeared to correlate with intracellular IFN-α production by NK cells (Table 3, Fig 2). However, this effect did not reach statistical significance due to the small number of samples.

The results of the ICS assay showed that NK cells produced IFN-α in response to IL-29 stimulation alone although this secretion was not detectable in the supernatant assay (Table 3). Possible explanations for this discrepancy included the different incubation times between the supernatant assay and ICS assay and the mixed cellular components in the culture conditions, which might have caused accelerated metabolism or use of the secreted IFN-α, if any, by the other cellular components in the culture.

The response of volunteer number 7 was distinct from those of the other volunteers, in that the intracellular IFN-α production by the monocyte fraction in response to IL-29 and IL-29 plus CpG stimuli were similar (Table 3). In this sample, the overall amount of IFN-α secretion by PBMCs in response to IL-29 plus CpG was similar to those of group C, despite the fact that the IFN-α secretion by PBMCs in response to CpG alone was comparable to those of group B (Fig 2). This unique result has provided insight toward a possible role of monocytes in PBMC responses to CpG or CpG plus IL-29, which merits further study. Although pDCs produced highest IFN-α levels per cell, monocytes were reported to be a competing IFN-α source in total PBMC due to their high frequency according to Hansmann et al. (24).

pDC intracellular IFN-α results should be notable since pDCs secret IFN-α 100~1000 times more than other PBMCs (8, 25). pDCs showed more intracellular IFN-α production in the CpG or CpG and IL-29 treatment groups than the control group. However, pDCs showed similar intracellular IFN-α secretion in the CpG or both CpG and IL-29 treatment groups, while supernatant IFN-α secretion doubled in both the CpG and IL-29 treatment groups. pDC IFN-α secretion could arise mainly after 4 hours of incubation with CpG-ODN or IL- 29. Particularly since IL-29 was considered a slow acting reagent in one mouse study (20), it could have an indirect effect after 4 hours of stimulation. It was not plausible in this study to directly correlate the supernatant IFN-α secretion with pDCs’s intracellular IFN-α production, so prolonged incubation for over 4 hours will be applied in the next study.

The ICS method was slightly modified from Tilton’s report (17) who reported a 6.15% increase in intracellular IFN-α production in the CpG treatment group compared to the control. The only difference between our study and Tilton’s study was the samples; we obtained samples from healthy donors and Tilton et al. (17) used HIV patients’ samples. pDCs from HIV patients could be regarded as being in a highly stimulated state compared to our healthy donor’s pDCs.

## Conclusion

IL-29 had an indirect enhancing effect with CpG on IFN-α secretion of PBMCs, while it alone did not induce IFN-α secretion of PBMCs. Given that pDCs are the major secretors of IFN-α in peripheral blood, this result has suggested the possibility that IL-29 will have an enhancing effect in IFN-α secretion of human pDCs. Although in this study, the supernatant IFN-α secretion did not directly correlate with pDCs’s intracellular IFN-α production, prolonged incubation of pDC and other PB subsets with CpG or IL-29 for over 4 hours could be applied in the future intra cytoplasmic flowcytometric studies. These studies will help to elucidate the action mechanism of IL-29 in human pDCs associated with viral infections.
